# The Effects of Imagination on Performance in Ballet: A Case Study

**DOI:** 10.3390/sports13050132

**Published:** 2025-04-24

**Authors:** Eisa Alokla, Maximilian Stasica, Martin Puttke, Vahid Firouzi, Maziar Ahmad Sharbafi, André Seyfarth

**Affiliations:** 1 Lauflabor Locomotion Laboratory, Institute of Sports Science and Centre for Cognitive Science, Technische Universität Darmstadt, Alexanderstraße 10, 64283 Darmstadt, Germany; 2 DANAMOS®—Dance Native Motion System, 10178 Berlin, Germany; 3 Control and Cyber-Physical Systems Laboratory, Department of Electrical Engineering and Information Technology, Technische Universität Darmstadt, Landgraf-Georg-Straße 4, 64283 Darmstadt, Germany

**Keywords:** mental images, focus of attention, ballet jump, range of motion, ground reaction force

## Abstract

Mental images such as foci of attention can significantly enhance the quality of movements, providing a positive effect on human performance, e.g., in dancers or athletes. Thirteen participants (height = 161 ± 13 cm, mass = 46.4 ± 17.3 kg, and age = 21 ± 8.4 years) with varying levels of experience in classical ballet were divided into three groups (amateur, professional, and children). Each participant performed three sauté en suite jumps, followed by an instruction to imagine “taking the floor with them” during the jump. The study aimed to assess the effect of this external focus on jumping performance using biomechanical modeling. Results showed a statistically significant increase in jump height and an expanded range of motion in the hip and knee joints after the intervention, suggesting a positive influence on movement quality. However, results varied among groups, with no significant change in leg stiffness across participants, though tendencies appeared within each group. These findings indicate that an external focus of attention could be a useful tool in dance pedagogy, enhancing performance quality across experience levels and supporting individual progress. The study recommends further research to explore the full impact of psychologically effective instructions on various aspects of physical performance.

## 1. Introduction

In recent years, the application of movement imagery in sports science has gained significant attention due to its potential to enhance motor performance. Movement imagery, the mental simulation of physical actions without actual movement, is believed to contribute to improvements in various athletic and artistic performances, including sports and dance. Ideokinetic training, a five-stage method, focuses on key elements of the movement rather than the full sequence. This reductionist movement analysis can induce a prevision of the imagination of the movement and can be verbalized. The movement itself can be corrected and optimized before execution within the verbalization [1]. Within the domain of classical ballet, jumping is a critical skill, and understanding how imagery can influence performance in this area is of great importance. The intersection of classical dance and scientific principles has historically been limited [2], as dance emphasizes expression and aesthetics, while science focuses on systematic observation and analysis. Movement imagery can be categorized into internal and external focus, both of which are thought to play crucial roles in motor performance. An internal focus involves concentrating on body movements, while an external focus shifts the attention towards a seemingly unrelated movement goal. This is to divert the attention away from the movement itself, allowing the body to realize a more natural movement [2]. The impact of these movement imagery types on the technique in classical dance remains underexplored [2,3,4].

Biomechanical tools, including motion capture and electromyography (EMG), offer new methods to analyze dance techniques, crucial for performance enhancement [5,6]. Comparative studies on joint range of motion (ROM) also demonstrate significant differences in dynamic balance between dancers and non-dancers [7]. Motor imagery practice (MIP) is a widely used method to enhance ballet performance, with evidence showing increased plantarflexion and ROM through mental simulations of movements [8,9]. Complementing this, studies like those using the Franklin method demonstrate how mental imagery can boost jump height [10]; however, this is not a primary concern in classical dance. Heiland et al. demonstrated the impact of imagination, specifically examining four mental images, on the performance of ballet dancers with a focus on increasing the average jump height assessed in [11]. This approach to mental and biomechanical training provides a holistic understanding of how movement and imagery can improve jumping performance, though gaps remain in addressing ROM in lower limb joints, which is crucial for assessing motor performance [12].

Different studies mentioned above demonstrated the effects of motor imagery on movement performance, but the evolution of body parameters to shape new movement dynamics was not investigated. A potential solution to fill this gap of knowledge is developing biomechanical models to identify changes in human motor control based on an external focus. However, the complexity of the human musculoskeletal system and neural control makes understanding motor imagery’s effects on motor control and its results on performance very challenging. The template and anchor concept [13] introduced to enable understanding the functionality of detailed models (termed as “anchor”) using abstract representation (termed as “templates”) could offer insight into the presented problem, but they have not been practically implemented by practitioners in dance. One of the basic template models is the spring-loaded inverted pendulum (“SLIP”) [14], which focuses on leg functioning in locomotion. We hypothesize that using such template-based modeling, which can retain the key biomechanical features of human locomotion, may reveal new insights to realize the effects of motor imagery on ballet dance performance.

Biomechanical studies on human locomotion showed three basic locomotor sub-functions are sufficient for generating different gaits [15,16]. In [17], Puttke proposes the idea of seven basic units of human movement, which he derived from everyday human actions. These units are called *morphemes,* which is a term taken from linguistics that describes the smallest meaningful unit of a given context. In the field of movement, this, therefore, describes key movements that cannot be separated any further without losing their underlying meaning. While these morphemes originate from general human motion, they also serve as foundational elements in the technique of every form of artistic dance [17]. Concerning this, he builds the DANAMOS®-Dance.native.motion.system concept to expand traditional dance didactics.

**Figure 1:**
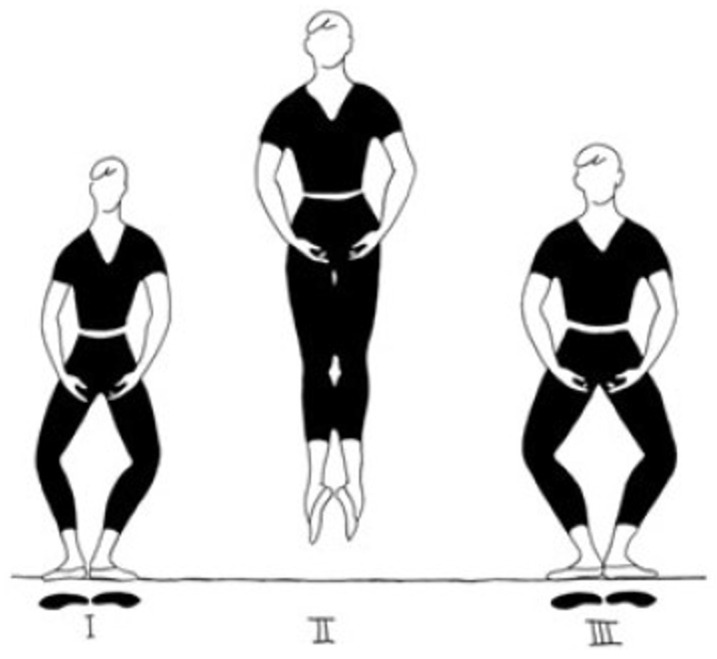
A sketch of the Sauté jump. In the preparation phase (**I**), the dancer goes into a plié from first position. During the flight phase (**II**), the body is straight and ideally stable. The landing (**III**) has to be performed in the same posture as the pre-flight phase. Below the dancer, the foot positions are outlined separately (own work, adapted from [18]).

This concept underscores the importance of innovative teaching strategies that align more closely with natural human motion [16]. As part of this concept, he developed an external focus of attention designed specifically to enhance jumping techniques in classical dance [17]. During jumping, energy should only be transferred to the CoM of the body, independent from all upper-body movements. The proposed mental image, “Take the floor with you!”, is supposed to encourage this beneficial energy transfer by adding a functional meaning to the jump [17]. This is supposed to lead to multiple improvements in jumping technique. In this study, the aforementioned external focus of attention was utilized to provide a functional meaning to the jump of Sauté. This jump is one of the basic elements of the jumping technique in classical dance, which consists of over 140 jumping variations [18] and involves the dancer jumping vertically into the air while starting and landing in first position (see Figure 1). We hypothesized that the effects depend on the participants’ experience level in dancing and their learning abilities. To investigate these two factors, we considered three groups of amateur, professional, and child participants. We expected that focusing on leg function using the SLIP model could explain motor imagery and external focus influences on dance performance in all participants, while these effects may vary across different groups.

To our knowledge, despite the increasing interest in motor imagery within sports science, its application in classical ballet remains an underexplored area. While previous studies have examined the influence of imagery on performance [8,9,10], few have investigated how external focus imagery specifically affects biomechanical and neuromuscular adaptations in ballet jump execution. Given the complex interplay between motor control, coordination, and artistic expression in dance, this research addresses a critical gap in the literature by integrating biomechanical modeling to assess the effects of external focus on ballet performance. This study also examines the effects of experience levels (amateurs, professionals, and children) on responding to external focus cues for optimizing jump techniques, which need further investigation in the future.

Using the SLIP model, we focus on the “stance” as the first locomotor sub-function and a crucial one for vertical jumping, which is the focus of this study.

## 2. Materials and Methods

### 2.1. Experiment

The experiment involved thirteen (12 female and 1 male) healthy young participants (mean ± SD: height = 161 ± 13 cm and mass = 46.4 ± 17.3 kg; age = 21 ± 8.4 years). As detailed in Table 1, participants were divided into three groups based on age and ballet dancing experience. First group: A group of students with varying levels of expertise in ballet dancing (amateur group). Second group: Participants with strong experience in ballet dancing, e.g., members of a professional ballet group (professional group). Third group: Child participants with varying experiences in ballet dancing, ranging from good to quite simple experience. These children were recruited from a local ballet school (child group). Participants were selected based on the following criteria: (1) basic to advanced experience in ballet dancing, (2) no lower limb injuries in the past six months, and (3) the ability to perform repeated jumps without excessive fatigue. Participants with any musculoskeletal disorders were excluded. The study was conducted in accordance with the Declaration of Helsinki and approved by the Ethical Board of TU Darmstadt (EK 47/2021).

The experiment was guided by an experienced professional dance teacher. Initially, participants were instructed to perform three Sauté en suite, that is, consecutively, using their previously learned technique. Subsequently, they received the external focus of attention, “Take the whole floor with you”, which was presented orally. Here, this instruction was used to direct dancers’ attention to an external focus. An external focus refers to concentrating on the intended effect of the movement on the environment, such as imagining the floor movement within the jump. This contrasts with an internal focus, where the attention is directed toward the body’s movements, such as thinking about the contraction of muscles during the jump. By promoting an external focus, the instruction aims to enhance the dancer’s performance, leading to greater jump heights and improved stability. This was phrased in this particular way to provide meaning (“lifting”) to the Sauté jump, which was theorized to result in larger forces being exerted to achieve higher jumping height. Further, this instruction is thought to facilitate the stabilization of the body axis. After the instruction, the participants repeated the Sauté en suite three times.

### 2.2. Modeling and Analysis

We used the plug-in gait model with a total of 36 markers, covering both the upper and lower limbs. The markers were placed on joint centers and bony landmarks, ensuring accurate kinematic measurements. Additionally, tracking markers were positioned on the limbs to enhance motion capture precision. Joint kinematics and angles were recorded using 12-camera Qualisys Oqus motion capture at a sampling rate of 460 Hz (Qualisys Inc., Gothenburg, Sweden), ensuring high-precision tracking and minimizing errors in dynamic motion recording. The system was calibrated accordingly before data collection.

**Figure 2:**
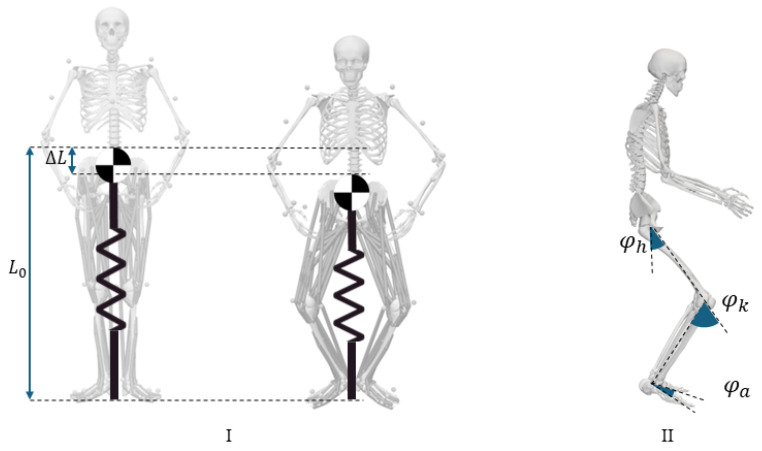
(**I**) The spring-loaded inverted pendulum representing the behavior of CoM. $L_{0}$ represents the leg rest length, and ΔL indicates the change in the spring length compared to $L_{0}$. (**II**) Sagittal view of the human model showing the definition of hip ($\psi_{h}$), knee ($\psi_{k}$), and ankle ($\psi_{a}$) angles.

The analysis utilized a 12-segment, 29-degrees-of-freedom generic musculoskeletal model in OpenSim [19,20]. Figure 2 shows different jumping phases in the OpenSim model. The model was scaled to match each participant’s anthropometry based on experimentally measured markers on anatomical landmarks. Joint angles were calculated using an inverse kinematic (IK) tool. The IK output, together with the ground reaction force (GRF), was then used to compute the CoM [20]. Joint angles were calculated as the relative angles between adjacent segments of the body, specifically focusing on the hip, knee, and ankle joints during the takeoff and landing phases of the jump. The anatomical movements analyzed were flexion and extension in the sagittal plane. The definition of the hip, knee, and ankle joint angles is shown in Figure 2II. In the context of human movement and biomechanics, joint angles typically refer to the angular relationships between adjacent segments of the body. For this study, the anatomical movements referred to in Figure 2 are defined as follows.

Hip flexion/extension ($\psi_{h}$): the angle between the thigh and pelvis, where flexion refers to the movement bringing the thigh closer to the torso, and extension refers to the movement taking the thigh away from the torso.
Knee flexion/extension ($\psi_{k}$): the angle between the thigh and the lower leg. Flexion refers to bending the knee (reducing the angle), while extension refers to straightening the knee (increasing the angle).
Ankle dorsiflexion/plantarflexion ($\psi_{a}$): the angle between the foot and the shank. Dorsiflexion is the movement of the foot upward toward the shank, and plantarflexion is the movement downward away from the shank.

These angles are calculated using motion capture systems that track markers placed on bony landmarks of the body (see Figure 2), and the angles are determined using vector mathematics or kinematic equations based on these markers’ positions. To calculate rest length and stiffness, the spring-loaded inverted pendulum (SLIP) model [14] is used (see Figure 2). The leg length is calculated as the distance between the CoM and CoP. We applied linear regression to the vertical GRF and leg length to obtain a force–length curve during the stance phase. The stiffness coefficient (k) was extracted as the slope of the best-fit line on the force–length curve. The rest length ($L_{0}$) was calculated as the intercept of the fitted line and zero-force line [14,16]. This model simplifies the whole-body movement dynamics to a point mass at CoM on top of a massless spring and was frequently used for biomechanical analysis of human movement including jumping [21]. Representing the leg by a linear spring requires two values, rest length and stiffness, to characterize human leg function in the corresponding movement task.

By fitting a linear relation to the force–length curve, the stiffness and rest length of a virtual spring between CoM and CoP is calculated. The detailed steps (from [16]) are as follows.

1. Data collection: We measured the ground reaction force using force plates and determined the leg length (distance from the CoM to the CoP) using motion capture data.
2. Force–length relationship: The leg was modeled as a linear spring, where the force exerted by the leg (*F*) is related to the leg’s stiffness (*K*) and the difference between the rest length ($L_{0}$) and the actual leg length (*L*):
  $$F=K\cdot (L_{0}-L).$$
3. Linear regression: We performed linear regression on the force–length data collected during the stance phase. The slope of the resulting line provides the stiffness (*K*), and the intercept with the length axis (where *F* = 0) provides the rest length (*L*).

By fitting this linear model to the collected data, we directly obtained the values for leg stiffness and rest length without requiring further lookup or calculation steps.

For statistical analysis to determine significant differences between different conditions, since we utilized a within-subject design, we chose a linear mixed-effects model (LMM) to analyze the relationship between the biomechanical parameters in the pre- and post-condition. The statistical results, as shown in Table 2, indicate a clear trend in the data.

## 3. Results

**Figure 3:**
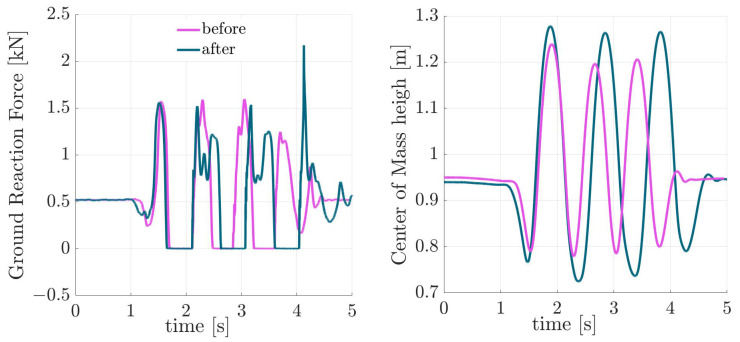
Exemplary graphs of the ground reaction force (GRF) and center of mass (CoM) height for one of the participants from the amateur group before and after imagination. The graphs illustrate increased leg compression and higher jump height after imagination, demonstrating improved force absorption and movement efficiency.

**Figure 4:**
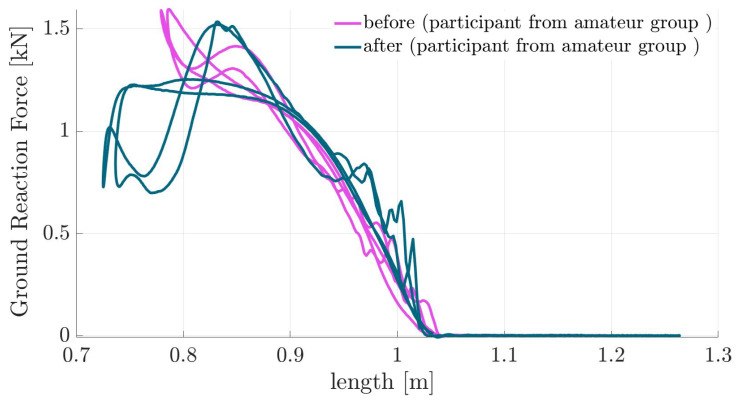
Interaction of the body with the ground through the leg, characterized by the force–length curve of the Sauté jump for one exemplary participant from the amateur group before and after imagination. This shows how external focus training modifies the interaction between the body and ground, leading to more effective energy utilization.

**Figure 5:**
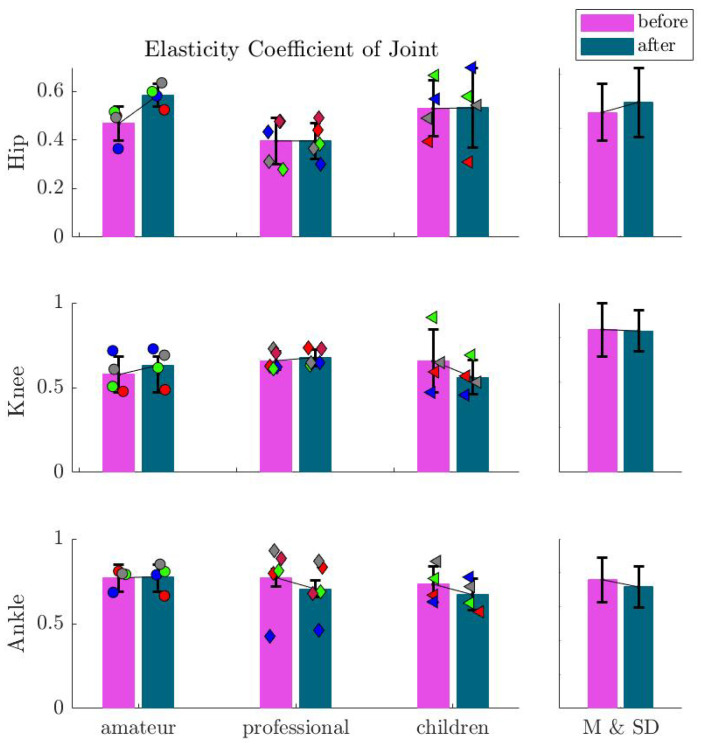
Mean and standard deviation of the elasticity coefficient of the hip, knee, and ankle joints (EC) before and after imagination. Markers show the values for each subject in the corresponding group.

**Figure A1:**
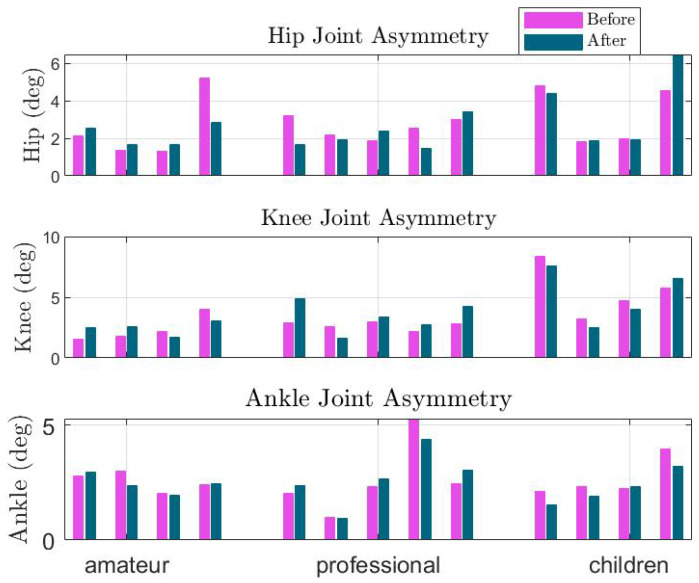
Asymmetry indices for the hip, knee, and ankle joints before and after imagination.

In this section, we present the kinematic and kinetic behavior before and after imagination. For each subject, we average the values of two hops. The asymmetry between the two hops is negligible (see Figure A1). Further, the interaction between the body and the ground is modeled with a springy leg, inspired by the SLIP model [14]. In Figure 3, the GRF and CoM heights are illustrated for one participant from the amateur group during three Sauté en suites before and after imagination. This shows typical behavior, demonstrating higher leg compression and increased jumping height after imagination. Exploring this behavior in the force–length curves in Figure 4 confirms enlarged work loops [22] after imagination. To analyze the changes in the work loop discussed in Figure 4, we use the elasticity coefficient, which is introduced to characterize energy shuffling at each joint [22]. Figure 5 demonstrates the effects of imagination on the elasticity coefficient at different lower-limb joints. A clear increase in hip elasticity for the amateur group and a decrease in ankle elasticity for professionals and children are observed, though not statistically significant.

### 3.1. Overall Behavior

**Figure 6:**
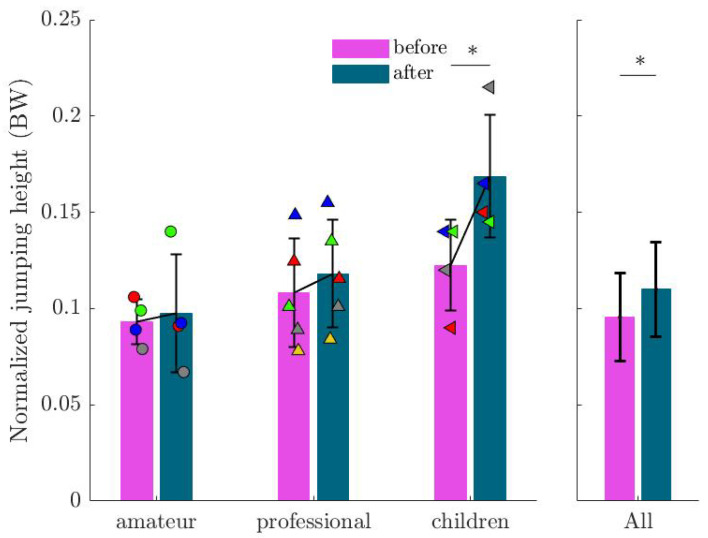
Mean and standard deviation (M and SD) for jumping height before and after imagination normalized by participants’ body weight. Markers show the values for each subject in the corresponding group. This highlights the positive effect of external focus, where 77% of participants showed an increased jump height. An asterisk (∗) indicates a statistically significant increase in jumping height (p<0.05).

A statistically significant increase in jumping height was observed in 77% of subjects (see Figure 6), with an average increase of 19% in jumping height after imagination. Statistical analysis confirmed the significance of this effect (β=0.0144; SE=0.0065; p=0.031).

**Figure 7:**
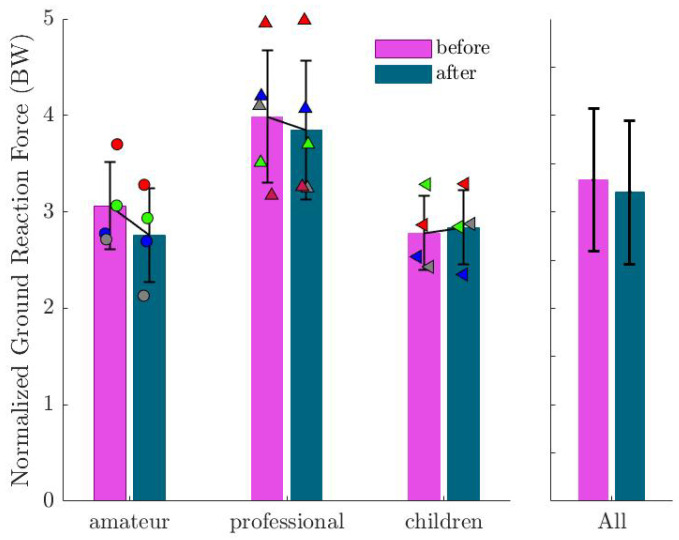
Mean and standard deviation of the vertical ground reaction force (GRF) before and after imagination normalized by participants’ body weight. Markers show the values for each subject in the corresponding group. This demonstrates a decrease in peak force for some groups, suggesting adjustments in movement mechanics.

Figure 7 depicts the GRF after normalizing the body mass. No significant difference was observed among all subjects, while the GRF decreased in all subjects of group 1. Figure 7 depicts the GRF after normalization, showing a decrease in the peak value for the first group after imagination, while there was no clear trend in groups 2 and 3.

To analyze leg behavior, we investigated the effects on rest length and stiffness of the best spring, which can predict leg force–length behavior.

**Figure 8:**
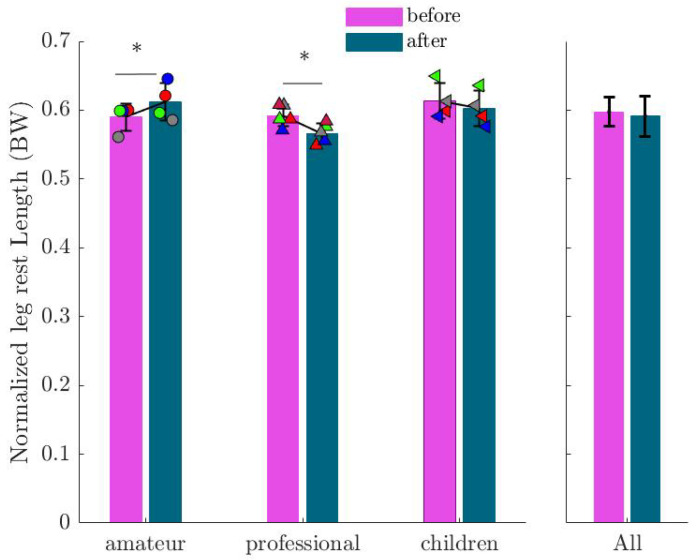
Mean and standard deviation of the identified leg rest length ($L_{0}$) before and after imagination normalized by participants’ body weight. Markers show the values for each subject in the corresponding group. An asterisk (∗) indicates a statistically significant increase in the leg rest length (p<0.05).

**Figure 9:**
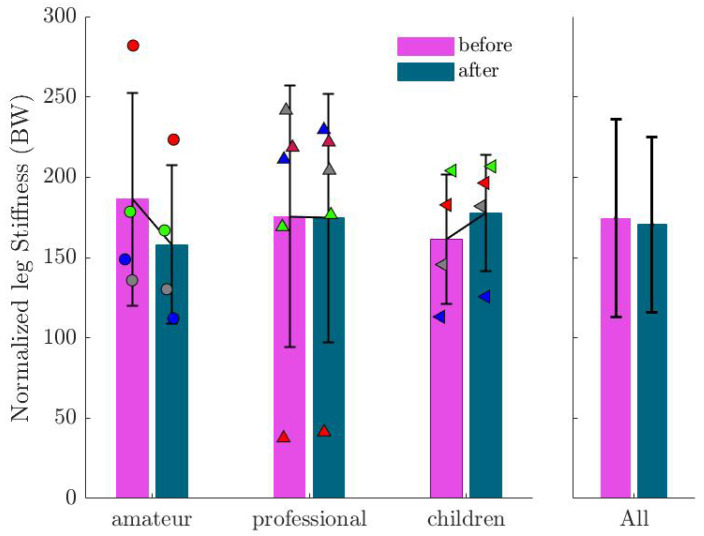
Mean and standard deviation for leg stiffness before and after imagination normalized by participants’ body weight. Markers show the values for each subject in the corresponding group.

Figure 8 presents the rest length ($L_{0}$) of the leg, where no significant change was found among all participants (β=0.0061; SE=0.0063; p=0.0332). Similarly, no significant difference was found in normalized leg stiffness across all participants (see Figure 9). However, trends of decreasing leg stiffness in the amateur group and increasing stiffness in the children group were noted, though not statistically significant.

**Figure 10:**
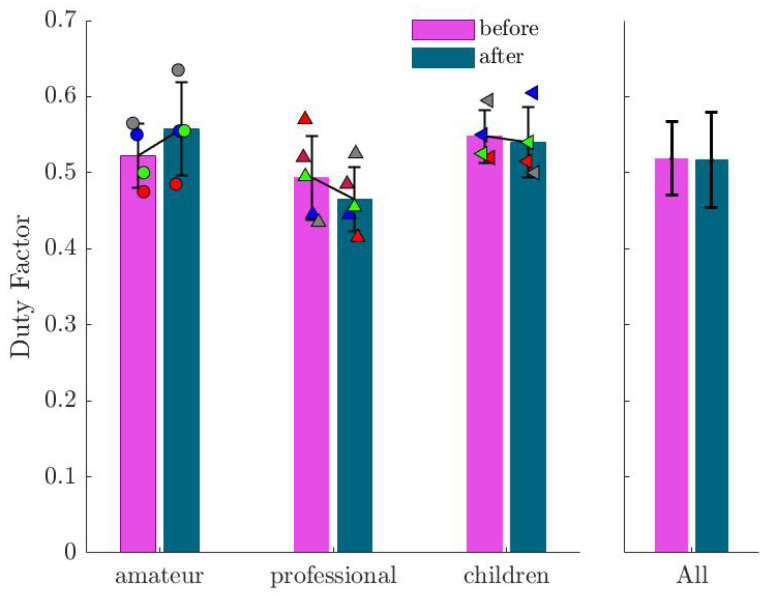
Mean and standard deviation of the identified duty factor (DF) before and after imagination. Markers show the values for each subject in the corresponding group.

The duty factor (DF), which represents the ratio between stance time and the whole jumping cycle, exhibited variations across individuals but no significant change when considering all subjects together (see Figure 10).

**Figure 11:**
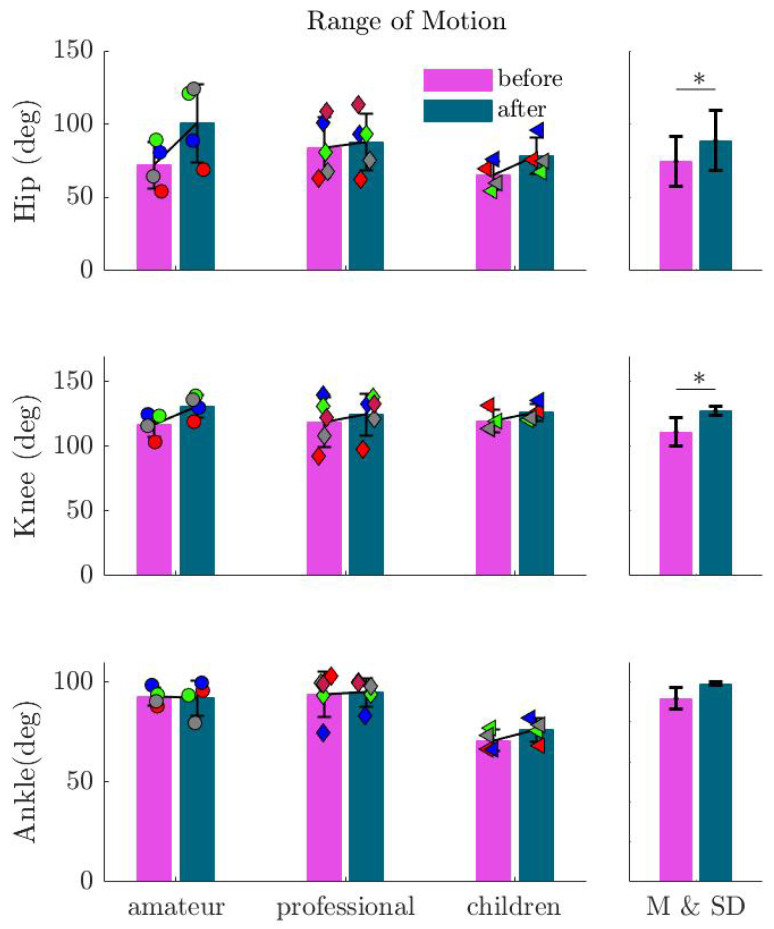
Range of motion in the hip, knee, and ankle joints (ROM) before and after imagination. Markers show the values for each subject in the corresponding group. This shows increased ROM in the hip and knee, particularly among children, confirming the role of imagination in enhancing flexibility. An asterisk (∗) indicates a statistically significant increase in the range of motion (p<0.05).

There was also no significant change in the grand mean over all subjects. Finally, we analyzed the changes at the joint level. It is clear from Figure 11 that there was a noticeable increase in the range of motion (ROM) in the hip and knee joints for all participants except one participant from the professional group. On average, the 21% increase in hip ROM (β=14.218; SE=5.1161; p=0.0076) and 8% increase in knee ROM (β=8.6265; SE=3.3511; p=0.01306) resulted from external focus. Conversely, there was no apparent trend in the ankle joint for all participants (β=1.9015; SE=1.9946; p=0.345).

**Figure 12:**
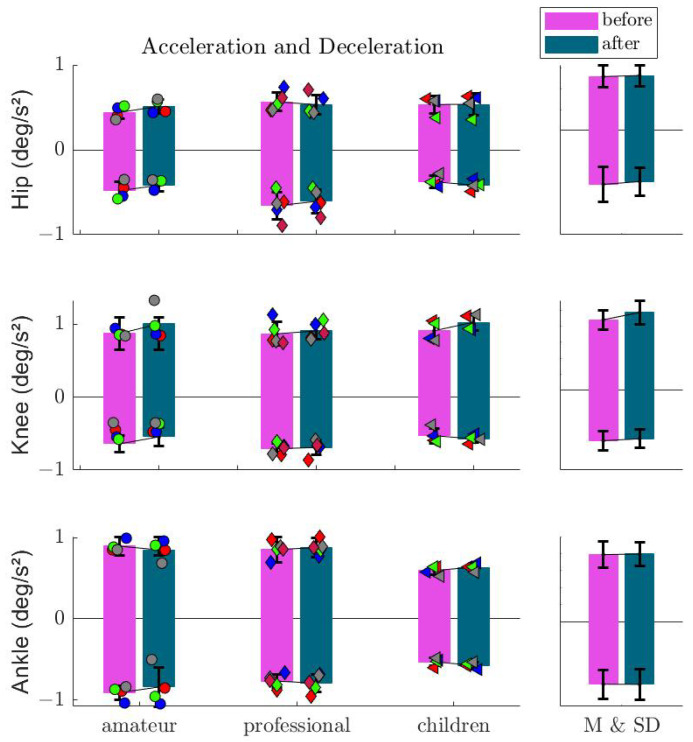
Mean and standard deviation of the acceleration and deceleration analysis of the hip, knee, and ankle joints before and after imagination. Markers show the values for each subject in the corresponding group.

Figure 12 shows the angular acceleration/deceleration at the hip, knee, and ankle joints before and after imagination. No significant change was observed in total or among different groups. The highest effect of imagination was observed at the knee joint for non-professionals.

### 3.2. Subgroup Behaviors

When examining individual group responses, distinct patterns emerged. Differences in leg elasticity and stiffness were observed among the groups before and after imagination. A significant reduction in rest length was found in one group (β=−0.0255; SE=0.0061; p=0.0006), while another group showed a significant increase (β=−0.0222; SE=0.0103; p=0.049). Trends in duty factor also varied among groups, with some showing an increase, others a decrease, and some exhibiting no clear trend. Variability in jumping height adaptations was also observed across groups, with different patterns in the redistribution of joint elasticity. Trends in the duty factor also varied among groups, with some showing an increase, others a decrease, and some exhibiting no clear trend. Variability in jumping height adaptations was also observed across groups, with different patterns in the redistribution of joint elasticity.

## 4. Discussion

Although the sample sizes in the subgroups are limited, increasing the number of participants would allow for more robust statistical analysis. Nevertheless, reviewing the current results and behavioral patterns provides valuable insights into individual differences in movement strategies, which can help shape future research. This study aimed to investigate the role of an external focus, introduced by instructions for imagination, before performing a specific jumping movement in ballet. Our outcomes revealed the significant influences on increasing jumping height and range of motion of proximal joints (hip and knee). In that respect, the results support the hypothesized effectiveness of imagination in improving jumping performance. Another finding is the identified difference between the learning effects in different subject groups. In the following, we elaborate on the findings from different levels of analysis.

### 4.1. Overall Behavior

Focusing on the force–length relation demonstrates a change in the leg behavior after imagination. As can be seen in Figure 3, the external focus results in longer hops, an emerging plateau region in the GRF, and an increase in CoM excursion. This reduces the elasticity of the leg while injecting more energy at the maximum compression position with elevated loading of the muscles. Lower elasticity can also be seen in the increased work loop in Figure 4. These changes in motor control result in an increased jumping height. To better analyze work loop changes with external focus among all participants, elasticity coefficients were demonstrated in Figure 5. We identified two main strategies of decreasing hip elasticity and increasing ankle elasticity, which are sometimes complemented with either an increase or decrease in knee elasticity. Since biomechanical parameters of ballet jumps are key to artistic perception and can even be used to partly predict perception by the audience [23], it might be advantageous to be able to shape the movement pattern with additional tuning features. In the case of ballet jumping, we found that after instructions, the force–length curve of human leg function adapted to a depression in leg force around mid-stance. Such an adaptation was also found in human racewalking [24]. Here, a similar depression in leg force at midstance was observed, which helped to prolong the contact phase (as flight phases are not allowed). In contrast, in artistic movements such as ballet jumping, this modification of the force–length curve could help support artistic expression. Thus, the force–length curve of the leg, as analyzed in human biomechanics, provides a fingerprint of the movement both in sports and arts.

The significant increase in jumping height (as a performance measure) among all subjects confirms the functionality of the external focus approach to improve dance performance, which is in line with previous studies [8,12,25]. External focus facilitates motor execution by directing attention away from the execution itself, reducing conscious interference with automatic motor control processes. Furthermore, external focus has been associated with enhanced neuromuscular activation patterns, as observed in studies demonstrating increased muscle efficiency and reduced co-contraction in complex motor tasks [8]. Notably, the external focus approach has been shown to benefit skill acquisition across different expertise levels, suggesting that even trained dancers can refine their technique and performance outcomes [12]. This evidence underscores the practical relevance of implementing external focus instructions in dance pedagogy, where subtle adjustments in attentional strategies can yield significant performance enhancements. It is worth mentioning that the changes in the shape of the GRF and elongation of the whole jumping time are not reflected in the average GRF and duty factor demonstrated in Figure 7 and Figure 10, respectively. Using neuromuscular modeling approaches, such as a sensor-motor map [26,27], could help better understand motor control to generate such behaviors. As imagination changes the elastic behavior of the leg (discussed in the work loop diagrams), simplifying the leg to a linear spring quantified by stiffness (K) and rest length ($L_{0}$) might not be sufficient to represent leg behavior. In other words, leg behavior will be less elastic if damping and energy injection are incorporated to enhance performance in safe execution [28].

Other aspects of achieving greater jump height, which are not reflected in our SLIP-based analysis in the sagittal plane, include movements of the lower extremities in the frontal plane and upper-body posture control. The observed variability in jump height among different groups may be attributed to their skill level, which determines how dancers control these additional factors. Differences in the neural control system and individual training backgrounds may be influencing factors on inter-subject (group) variability. Finding no consistent changes can be seen in k and $L_{0}$ in Figure 7 and Figure 8, which revealed that simplifying the leg behavior with linear spring in the sagittal plane, which also ignores work loop aspects, cannot describe the reasoning for performance enhancement. Advancement of the model by extending the SLIP model by adding upper-body and energy-management elements (like [29]) could be the next step to better predict imagination effects in human jumping control. Extending models to more advanced biomechanical (e.g., including neuromuscular details) levels in the future could improve our understanding of the underlying principles of movement control and the biomechanical and neural mechanisms involved [27].

### 4.2. Subgroup Behaviors

In addition to the general findings discussed before, focusing on the effects of imagination on different groups provides insights into the learning effects. Interestingly, the leg behavior (K and $L_{0}$) adaptation in professional and children groups is similar and different from the amateur group. For amateurs, external focus results in higher elasticity (equivalently smaller work loop) at both hip and knee joints. This means that amateur participants could find the instructions useful to increase their performance efficiency, although they do not increase their hopping height. Professionals just show a small decrease in ankle elasticity, which means a slightly larger work loop (more energy consumption), which results in a slight increase in hopping height. The largest changes are found in children who have reduced ankle and knee elasticity (consuming more energy), resulting in significantly higher hopping height. These more pronounced effects in children compared to amateurs and professionals likely relate to the higher responsiveness of children to mental stimulation [30], which enhances the dynamism of the muscles surrounding the joint and increases its flexibility.

Biomechanical analyses at the joint level could also help us to describe enhanced performance after imagination. The increased jumping height might be the result of an elevated range of motion in the proximal joints. The increase in ROM rises from distal to proximal joints. On the one hand, in the stance phase, increasing the ROM of distal joints (e.g., the ankle) needs much greater effort than proximal joints (e.g., the hips) due to the amount of body mass and inertia above the corresponding joints. On the other hand, the upper body holds a significant percentage of the body mass and inertia, and generating higher acceleration in the upper body in upward motion is sufficient to enhance the jumping height. Therefore, increasing the hip ROM is the most efficient way to increase performance. With the same argumentation, the next preferred joint will be the knee joint. These findings underscore the nuanced impact of imagination on joint flexibility, emphasizing its potential role in enhancing specific aspects of leg joint behavior.

It must be noted that this study only considered data from thirteen participants. A broader number of participants, at best from multicultural backgrounds, would be necessary to provide more far-reaching recommendations. This is especially important since the participating professional dancers were all recruited from the same ballet company; therefore, a similarity in previous experience and training must be assumed. It could be possible that professionals from other groups could react differently to the provided instruction.

### 4.3. Study Limitations

This study has several limitations that should be considered. First, the relatively small sample size may restrict the generalizability of the findings to a broader population. Second, the use of the SLIP model to simulate movement introduces certain simplifications that may not fully capture the complexity of the imagination effect on biomechanics. Lastly, focusing solely on kinematic and dynamic analyses may limit a deeper understanding of the underlying muscle mechanisms, which could be further explored through complementary methods such as electromyography (EMG) or muscle modeling approaches.

## 5. Conclusions and Future Work

In conclusion, this study delves into the intersection of biomechanics and imagination, specifically exploring the impact of an external focus of attention on the jumping performance of ballet dancers. The findings reveal a substantial increase in jumping height after imagination and confirm the hypothesis that instruction positively influences movement quality. This underscores the role of mental factors in enhancing ballet dancers’ performance and emphasizes the intricate interplay between physical and mental aspects in movement improvement.

Joint kinematics, angles, and ground reaction forces were scrutinized, unveiling changes in the force–length curve. The results indicate a clear positive impact on jumping height, which was particularly notable in the professional and child groups. Further exploration into leg stiffness, rest leg length, and duty factor demonstrated variations among participants but an overall positive trend in movement quality. To establish conclusive statements about the dance community as a whole, a larger sample size would be necessary, as the current sample is small, leading to an underpowered test. However, this study is the first step with a specific use-case introducing a new (template-based) methodology to analyze movement imagery effects on performance, which needs to be extended in the future.

The interdisciplinary nature of this research contributes to the understanding of how imagination techniques can be leveraged alongside biomechanics to enhance performance and movement quality. While most participants profited from the instruction, not everyone did. This highlights that there is no one-for-all approach in didactics and amplifies the importance of a qualified and experienced instructor. It is the task of the dance teacher to identify the individuals who most likely profit from such methods. This demonstrates that, although science can provide valuable insights and recommendations, interdisciplinary work with practitioners is the most valuable tool to support dancers in their careers.

Looking ahead, we aim to further explore the influence of instructions on movement quality. We plan to study the effects on the muscles of the lower limbs. Additionally, we intend to incorporate a control group in future studies. With this, we hope to broaden the horizons in improving the quality and performance of movement, especially among ballet dancers and athletes, through the use of psychologically effective instructions.

## Figures and Tables

**Table 1:** Demographic and physical characteristics (Mean ± SD) of dance groups by experience level.

Group	Sex	Age (Years)	Height (m)	Mass (kg)	Previous Experience
Amateur	4 f	23.8±6.6	1.63±0.08	38.3±21.8	Amateur Ballet
Professional	4 f, 1 m	26.8±5.1	1.69±0.06	59.4±8.2	Professional dancers
Children	4 f	11.0±1.7	1.38±0.10	38.2±13.0	Children’s ballet

**Table 2:** Summary of statistically significant results. This table presents the key statistical findings from the study, including β coefficients, standard errors (SE), and *p*-values. Statistically significant values (*p* < 0.05) are highlighted in bold.

Parameter	Group	β Coefficient	SE	*p*-Value
Jump Height Increase	All Subjects	0.0144	0.0065	**0.031**
Leg Rest Length Change	Group 1	−0.0222	0.0103	**0.049**
Leg Rest Length Change	Group 2	−0.0255	0.0061	**0.0006**
Hip Range of Motion (ROM)	All Subjects	14.218	5.1161	**0.0076**
Knee Range of Motion (ROM)	All Subjects	8.6265	3.3511	**0.01306**

## Data Availability

The data supporting the findings of this study are publicly available and can be obtained at https://doi.org/10.48328/tudatalib-1703.

## Abbreviations

The following abbreviations are used in this manuscript:

SLIP	Spring-Loaded Inverted Pendulum
ROM	Range of Motion
CoM	Center of Mass
CoP	Center of Pressure
GRF	Ground Reaction Force
LMM	Linear Mixed-Effects Model
